# Extreme Latent Solitary Lung Metastasis of Endometrial Carcinoma 23 Years Following Radical Treatment: A Case Report

**DOI:** 10.7759/cureus.67692

**Published:** 2024-08-24

**Authors:** Nilanthi Gunathilaka, Seemali Hannagala Gamage, Saman Kularatne

**Affiliations:** 1 Pulmonology, National Hospital for Respiratory Diseases, Ragama, LKA

**Keywords:** case report, endometrial carcinoma, pulmonary metastasis, solitary, latent

## Abstract

Female genital tract tumors are an infrequent cause of secondary pulmonary metastases. Endometrial carcinoma (EC) has a propensity to cause metachronous lung metastasis with an average three-year interval of the radical treatment for EC. We present the case of a patient with hemoptysis who had an isolated right middle lobe lung metastatic adenocarcinoma of the endometrium 23 years following radical hysterectomy for International Federation of Gynecology and Obstetrics (FIGO) stage 1 EC.

## Introduction

Female genital tract tumors are rare to cause pulmonary metastasis. However, the most common extra-pelvic destination of metastasis of those tumors is the lungs [1]. Most common female genital tract carcinomas are of endometrial origin and mostly cause recurrence [2]. The incidence of solitary lung metastases of primary endometrial carcinoma (EC) is nearly 2.3% to 7% [3]. Eighty percent of these metastases occur within three years of average latency [4].

The longest latency of pulmonary metastasis reported in the literature is 17 years after the curative treatment [5]. We report a case of an isolated pulmonary metastasis in a patient who was treated by hysterectomy and bilateral salpingo-oophorectomy 23 years prior.

## Case presentation

A 72-year-old female patient, postmenopausal for 23 years, was treated in May 2000 for an EC, which was discovered following evaluation for heavy menstrual bleeding. Contrast-enhanced computed tomography (CECT) abdomen and pelvis revealed endometrial hyperplasia without the distant spread of the tumor. The patient had undergone total abdominal hysterectomy with bilateral adenexetomy which revealed a well-differentiated papillary adenocarcinoma involving the entire endometrial cavity and endometrial canal without myometrial invasion (International Federation of Gynecology and Obstetrics (FIGO) stage 1). Following the diagnosis, the patient had been successfully treated with chemotherapy and radiotherapy.

After 23 years, in November 2023, the patient presented with on-and-off scanty hemoptysis and weight loss for three months duration. Until this presentation, the patient was stable. Additionally, she has diabetes, hypertension, and a recent myocardial infarction six months ago. Her performance status was stage 2. Her clinical examination was unremarkable.

**Figure 1:**
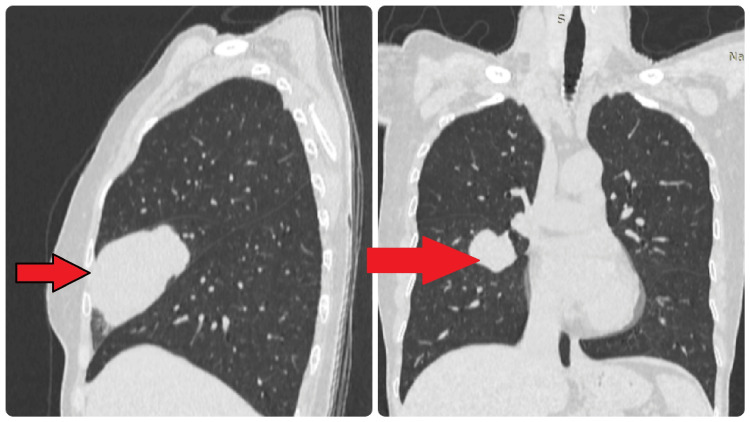
Contrast CT images indicate well-defined, isolated metastatic deposits in the right middle lobe (red arrows).

The chest X-ray showed a right middle lobe with large solitary opacity. A CECT of the chest revealed an irregular heterogeneously enhancing mass of 5.8 cm x 6.2 cm x 6.2 cm in size without local or distant metastasis (Figure 1). The tumor staging was T3N0M0. The bronchoscopic evaluation was normal without endobronchial invasion of the tumor. Due to the unavailability of the resource, we did not perform positron emission tomography (PET) for further assessment.

Biopsies of image guidance were consistent with metastatic adenocarcinoma, which had strong estrogen receptor (ER) positivity and strong vimentin positivity in the cytoplasm of tumor cells. Furthermore, TTF-1 was negative in the tumor cells. Histology is suggestive of metastatic adenocarcinoma from previously known endometrial carcinoma.

Though the lung metastasis was operable, the patient was not a suitable candidate to undergo major surgery due to the prevailing cardiac comorbidities; hence, palliative chemotherapy was planned after discussion with the lung cancer multidisciplinary team.

## Discussion

Common malignant neoplasms of uterine origin are endometrial adenocarcinoma, uterine sarcoma, and epidermoid carcinomas. Endometrial carcinoma is the most frequent neoplasm in the female genital tract and is an infrequent cause of pulmonary metastases. Of them, 80% are metachronous and 20% are synchronous [1, 6]. Five-year survival after curative surgical resection of the pulmonary metastases is variable between 32% and 46% [7, 8]. Being a unilateral lung metastasis, a disease-free interval (DFI) of more than 24 months, grade 1-2 histology, ER positivity, tumor size less than 2 cm, and less than 50% myometrial invasion are associated with a good prognosis [6]. Factors of poor prognosis are bilateral lung disease, DFI of less than 24 months, and metastases with size greater than 2 cm [9].

Falkenstern et al. reported isolated latent pulmonary metastasis following 14 years and 17 years of latency of a primary endometrial carcinoma [5]. Kodia et al. reported a case of a 20-year latency between hysterectomy for endometrial adenocarcinoma and solitary pulmonary metastasis [10].

We present a case of a remote, isolated pulmonary metastatic lesion appearing 23 years after the primary EC resection and curative treatment. After analyzing the patient’s symptomatology and extreme history of endometrial carcinoma with imaging evidence, either primary lung carcinoma or lung metastasis was entertained in the differential diagnosis. However, histology and immunohistochemistry showed negative TTF-1 and strongly positive vimentin staining and ER expression. TTF 1, being highly sensitive (84%) and highly specific (85-100%) for reliably identifying primary lung adenocarcinoma, ruled out the possibility of primary lung malignancy in this patient [11]. Vimentin-positive staining and ER positivity strongly suggested the high likelihood of a secondary tumor originating from the previously known EC.

Options for isolated pulmonary metastases are metastatectomy, chemotherapy, radiotherapy, or hormone therapy. Out of the above, metastatectomy is safe and provides favorable long-term outcomes in patients who have DFI of more than 24 months [12]. Five-year survival after resection of isolated pulmonary metastases from endometrial cancers is 69% [12]. However, no data mention the benefit of post-resection chemotherapy or hormone therapy [12, 13].

In summary, to the best of our knowledge, this case describes the extreme latent presentation of secondary pulmonary metastasis 23 years following curative treatment for primary EC. 

## Conclusions

Endometrial adenocarcinomas, being the most common uterine neoplasms, can manifest as infrequent pulmonary metastases. The majority of secondary metastases of a known EC are metachronous in origin. The average latency of presentation is three years. However, patients can develop extreme latent pulmonary metastatic disease as a manifestation of primary EC even after 23 years. The majority of such tumors can be treated with surgical resection and have a good long-term outcome. Therefore, regular monitoring of patients with symptom analysis, clinical examination, closer follow-up, and chest imaging are of utmost importance.
